# Feticide Before Termination of Pregnancy in Singleton Pregnancy – Trends in England and Wales 2012–2020, a Cross-sectional Study

**DOI:** 10.1007/s43032-023-01352-3

**Published:** 2023-09-25

**Authors:** Isabelle Schiff, Panicos Shangaris, Mary Grinsted, Srividhya Sankaran

**Affiliations:** 1 GKT School of Medical Education, King’s College London, Guy’s Campus, Great Maze Pond, London, UK; 2 Peter Gorer Department of Immunobiology, School of Immunology and Microbial Sciences, Guy’s Campus, Great Maze Pond, Faculty of Life Sciences and Medicine, King’s College London, London, United Kingdom; 3 School of Life Course and Population Sciences, King’s College London, 10th Floor North Wing St Thomas’ Hospital, London, London UK; 4 Fetal Medicine Research Institute, King’s College Hospital, London, UK; 5 Department of Women and Children, Guy’s and St Thomas’ NHS Foundation Trust, London, UK; 6 Department of Health and Social Care, London, UK

**Keywords:** Feticide, Termination of pregnancy, Singleton pregnancy, Health and Social Act 4 (HSA4), Department of Health and Social Care (DHSC), Potassium chloride, Abortion Act 1967

## Abstract

Feticide is the practice of inducing fetal demise before the termination of pregnancy. In England and Wales, it is recommended for terminations of pregnancy beyond 21+6 weeks of gestation. This project analyses the trends in feticide in singleton pregnancy in England and Wales between 2012 and 2020. This project was a retrospective study that analysed data extracted from the Health and Social Act 4 (HSA4) forms submitted to the Department of Health and Social Care (DHSC). The data extracted by the DHSC included the prevalence of feticide, methods of feticide and termination, statutory grounds, gestation, service provider, maternal age, ethnicity and obstetric history. In addition, data analysis was carried out to identify trends. Between 2012 and 2020, there were 9310 feticides in England and Wales, undertaken in 0.5% of all abortions. The prevalence of feticide fluctuated; however, there was an overall decrease from 1084 cases in 2012 to 1000 cases in 2020. Intracardiac injection of potassium chloride was the most frequent method of achieving feticide (67.2%). Just over half (55.8%) of feticides took place under Ground E of the Abortion Act 1967, with the main indication being congenital malformations of the nervous system. Two-fifths (40.2%) of feticides took place at 23 weeks, 22.8% at 22 weeks and 13.5% between 20 and 21 weeks. The remainder occurred at later gestations: 17.5% at 24–29 weeks and 5.9% beyond 29 weeks. During our study period, it was more common for feticides to be carried out as part of a medical termination than a surgical termination and 60.3% occurred in NHS hospitals. Women undergoing feticide were mostly aged 30–34 years (38.3%) and of White ethnicity (78.6%). Feticide is an essential component of comprehensive abortion care for women undergoing late second and third-trimester abortions. This study provides insight into how feticide is carried out in England and Wales and demonstrates the effect of the COVID-19 pandemic on reducing feticide prevalence. Future research should analyse in more detail the use of the different methods of feticide.

## Introduction

About 73 million terminations of pregnancy (TOPs), also known as abortions, are carried out worldwide every year, and comprehensive abortion care is deemed an essential healthcare service [1]. In the UK, less than 1% of abortions take place beyond 22 weeks. Feticide is a medical procedure that forms part of the recommended care for these later TOPs as it is used to induce fetal demise in the uterus, preventing livebirth in the context of abortion.

Under the Abortion Act 1967, abortion is legal in the UK (excluding Northern Ireland) for up to 24 weeks or in exceptional medical circumstances beyond 24 weeks. Grounds C and D of the Abortion Act cover TOPs that occur prior to 24 weeks due to risk to the physical or mental health of the pregnant woman or her existing family. Ground E is used when there is a substantial risk that if the child were born, it would suffer from physical or mental abnormalities as to be seriously handicapped. Under Ground A, abortion is legal at any gestation to save the life of the pregnant woman and under Ground B to prevent grave permanent injury to the physical or mental health of the pregnant woman. Grounds F and G are used for the same reasons as Grounds A and B, respectively, but in emergencies only [2].

The Royal College of Obstetricians and Gynaecologists (RCOG) recommends that feticide be routinely offered to patients who have decided to undergo a TOP after 21+6 weeks [3]. TOP resulting in a live birth is rare before 22 weeks of gestation. However, after this time, the chances of having a live birth following TOP increase [3]. Feticide is therefore carried out to avoid this by inducing fetal demise in the uterus before TOP.

Abortion providers use feticide before TOP for several reasons. Successful feticide prevents the birth of a neonate with signs of life, which can be distressing and potentially traumatising for patients and healthcare providers. Stillbirth is often preferred to livebirth neonatal palliative care by parents and healthcare staff involved in terminations [4–6]. In some settings, this may also reduce the potential risk of legal action against healthcare professionals in these situations, which is a concern for the staff involved [7, 8]. Additionally, there is some evidence that feticide is associated with a decreased duration of the TOP procedure [9–11].

Feticide before abortion of singleton pregnancy was first introduced in the 1990s and is now used routinely in late TOPs. Changes in the law and medicine increased the role of feticide in abortion care. In 1990, changes to the Abortion Act by the Human Fertilization and Embryology Act allowed TOP for severe fetal abnormality without a gestational limit. Advances in prenatal fetal imaging and fetal medicine also improved the ability to diagnose fetal abnormalities [4]. Many abnormalities are only diagnosed later in pregnancy, most often at the 20-week fetal anomaly scan. Given the time taken to get specialist opinions, to decide to terminate the pregnancy and to organise the procedure, these TOPs may occur after 22 weeks when the use of feticide is recommended.

Most TOPs in England and Wales are performed under Ground C of the Abortion Act (permitted up to 24 weeks) due to maternal anxiety or depression. Therefore, TOPs after 22 weeks under Ground C of the Abortion Act also require feticide in the termination of viable pregnancies without fetal abnormality. The age of viability has also decreased, with an increasing number of babies born at 22–24 weeks surviving [5, 6].

There are several methods of feticide used worldwide. The current method recommended by the RCOG to guarantee fetal asystole is intracardiac potassium chloride (KCl) [3]. Although this is the recommended method, other methods are mentioned in the literature and are sometimes used in clinical practice. These include umbilical KCl, intra-amniotic or intrafetal digoxin, and umbilical or intracardiac lidocaine. Additional methods of feticide are mentioned in this study, including intra-amniotic, extra-amniotic and umbilical urea, cordotomy and laser. However, these are rarely used, and there is very limited literature on these methods.

## Aims and Objectives

This study aims to identify the trends in the use of feticide before the termination of singleton pregnancies in residents and non-residents of England and Wales between 2012 and 2020.

## Materials and Methods

This is a retrospective observational study looking at the trends in feticide prior to TOP in singleton pregnancy in England and Wales, in collaboration with King’s College London and the Department of Health and Social Care (DHSC). This study will determine the prevalence of feticide in the study period and the types of feticide methods. Other variables will also be analysed, including method of termination, statutory grounds, Ground E diagnoses, gestation, provider, maternal age, ethnicity and obstetric history.

### Study Population

The information in this study was collected from HSA4 notification forms submitted to the Chief Medical Officers of England and Wales (CMO) by practitioners at clinics and hospitals in accordance with the Abortion Act 1967. For this study, the DHSC collected annual data from HSA4 forms where a feticide was performed or the gestation indicated that a feticide should be performed in residents and non-residents of England and Wales from 2012 to 2020. All residents and non-residents who underwent a confirmed feticide in this time period were included in the data analysis. Variables were collated and aggregated in a Microsoft Excel database. Approval from the Research Ethics Committee or CMO was not required as no patient-level data was used. This data was then analysed to collate and compare variables over the study period. Information from the DHSC-published abortion statistics over the same period was also analysed and commented on in this study.

Data on the method of feticide in 2012 and data on the method of feticide for non-residents in 2013 were not stored, and so these years were not included in the analysis of feticide methods. Data on the Ground E diagnoses from 2019 and 2020 was also excluded as it had all mentions of diagnoses rather than the primary diagnosis as in previous years. Thus, it could not be accurately combined and compared with data from other years. Multiple pregnancies with selective feticide were also excluded in all years. Due to the small number of feticides performed using cordotomy, intraamniotic KCI/urea, umbilical KCl/urea, extra amniotic KCl/urea, or laser and the confidentiality risks associated with this, these methods have been grouped. They were grouped differently in different years and, thus, had to be grouped as a whole in the analysis. Each year, they are grouped to control for disclosure because there were between 1 and 5 in each of these totals.

## Results

### Prevalence

Between 2012 and 2020, there were 1,776,491 abortions reported in England and Wales. In the same period, 9310 feticides were reported. Thus, only 0.5% of abortions were preceded by feticide.

**Fig. 1:**
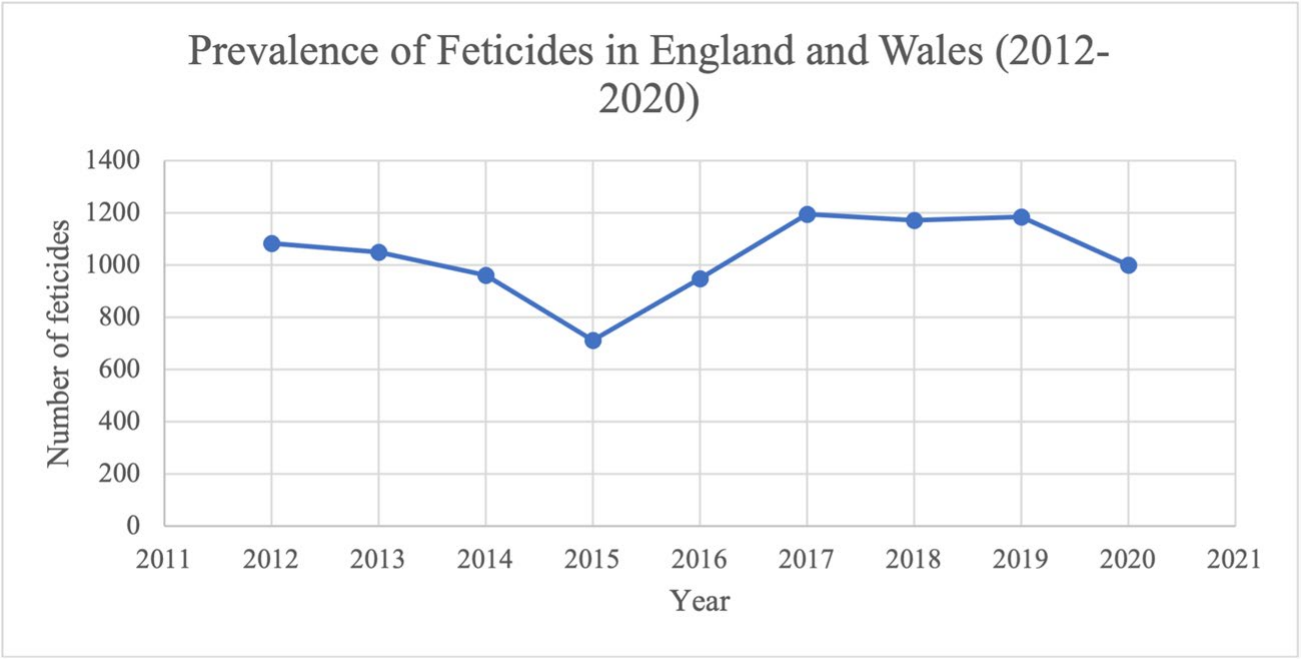
Trend over time of the number of feticides that took place in residents and non-residents of England and Wales between 2012 and 2020. Data from the DHSC

Overall, the number of feticides has fluctuated over the study period of 2012–2020 (Fig. 1). The total cases decreased by 7.8% over the study period, from 1084 in 2012 to 1000 in 2020. There was a reduction between 2012 and 2015, with a minimum number of cases of 712 in 2015. This was followed by a steady increase between 2015 and 2017, reaching a peak of 1195 feticides in 2017. Between 2017 and 2019, the number of cases remained relatively stable. Between 2019 and 2020, there was a decrease of 184 cases (15.5%).

### Method of Feticide

**Fig. 2:**
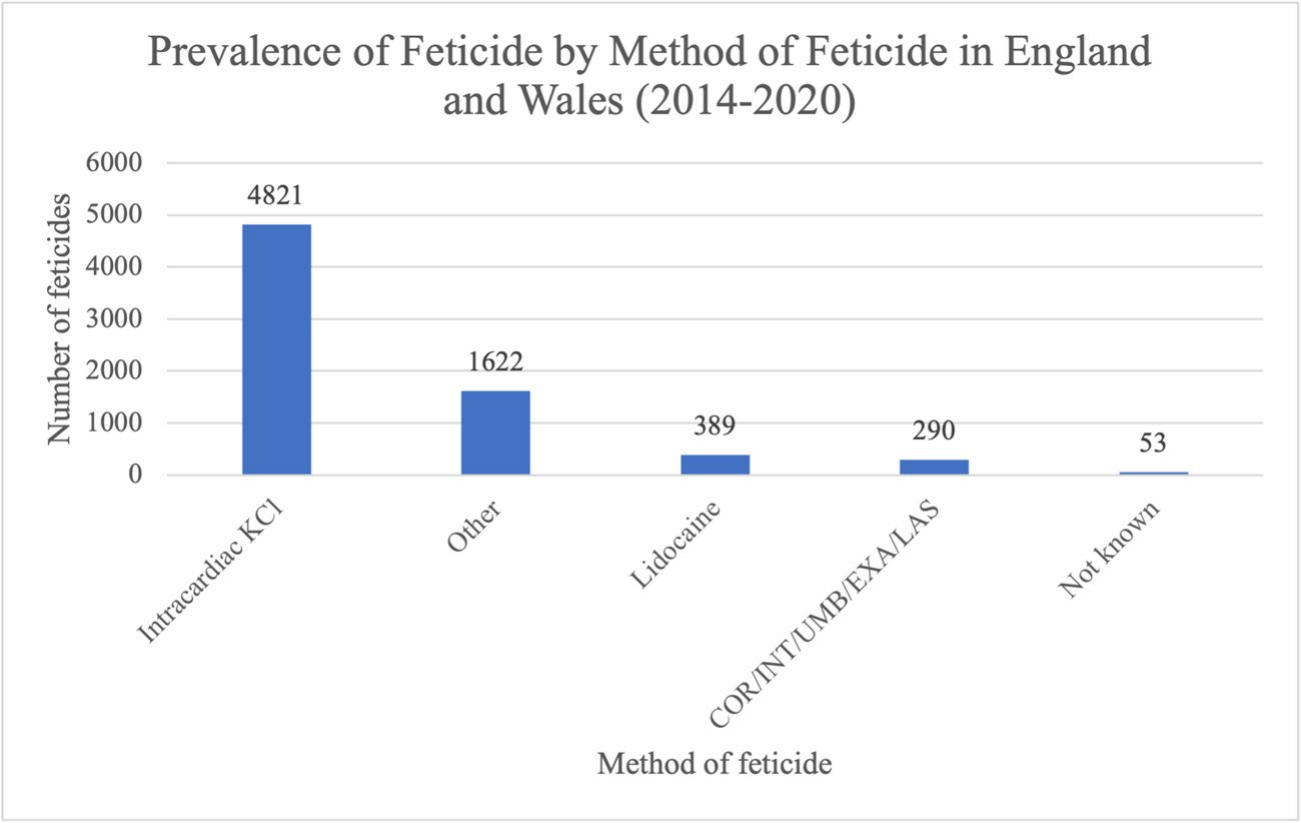
Prevalence of feticides by method/agent of feticide in residents and non-residents of England and Wales between 2014 and 2020. Data from the DHSC

Intracardiac KCl was the most prevalent method used to induce fetal demise before TOP in 2014–2020 (Fig. 2); 4821 feticides (67.2%) were performed using intracardiac KCl. The method of feticide for 1622 feticides (22.6%) was coded as other. This includes methods such as digoxin, transplacental administration and cordocentesis; 389 feticides (5.4%) were carried out using lidocaine; however, the site of administration was not recorded. In addition, 290 feticides (4.0%) were carried out using either cordotomy (COR), intraamniotic KCl/urea (INT), umbilical KCl/urea (UMB), extra amniotic KCl/urea (EXA) or laser (LAS). In 53 cases (0.74%), although it was known that feticide was performed, the method of feticide was reported as not known.

### Method of Termination

**Fig. 3:**
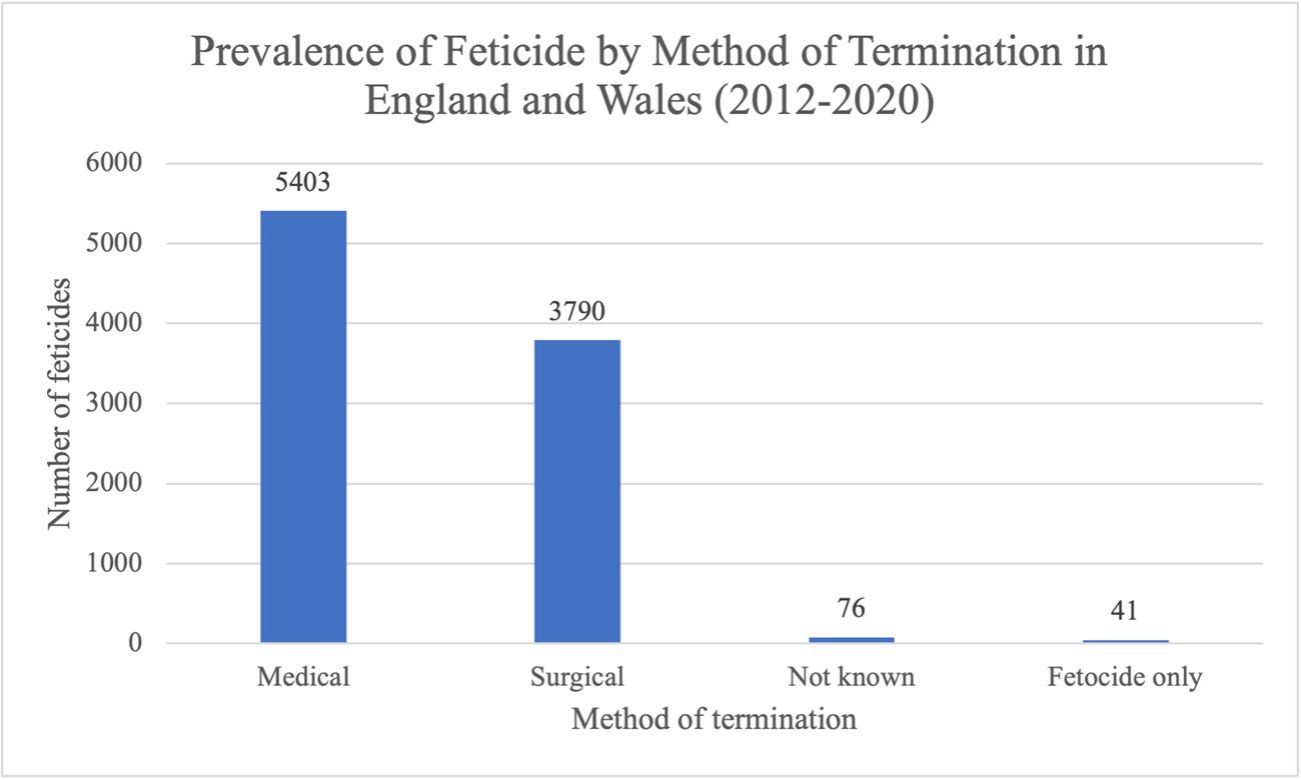
Prevalence of feticides by method of termination in residents and non-residents of England and Wales between 2012 and 2020. Data from the DHSC

During our study period, more feticides were carried out as part of a medical termination than a surgical termination (Fig. 3); 5403 feticides (58.0%) that took place were part of medical terminations, compared to 3790 feticides (40.7%) which were part of a surgical abortion procedure. In 76 cases (0.8%), although it was known that feticide took place, the method of termination was not known. For 41 cases (0.4%), the method of termination was recorded as feticide only. These are cases where the feticide occurred in England or Wales, but the rest of the termination procedure occurred in another country. Thus, all 41 of these patients were non-residents, with 40/41 residents of places outside of the UK.

### Statutory Grounds

**Fig. 4:**
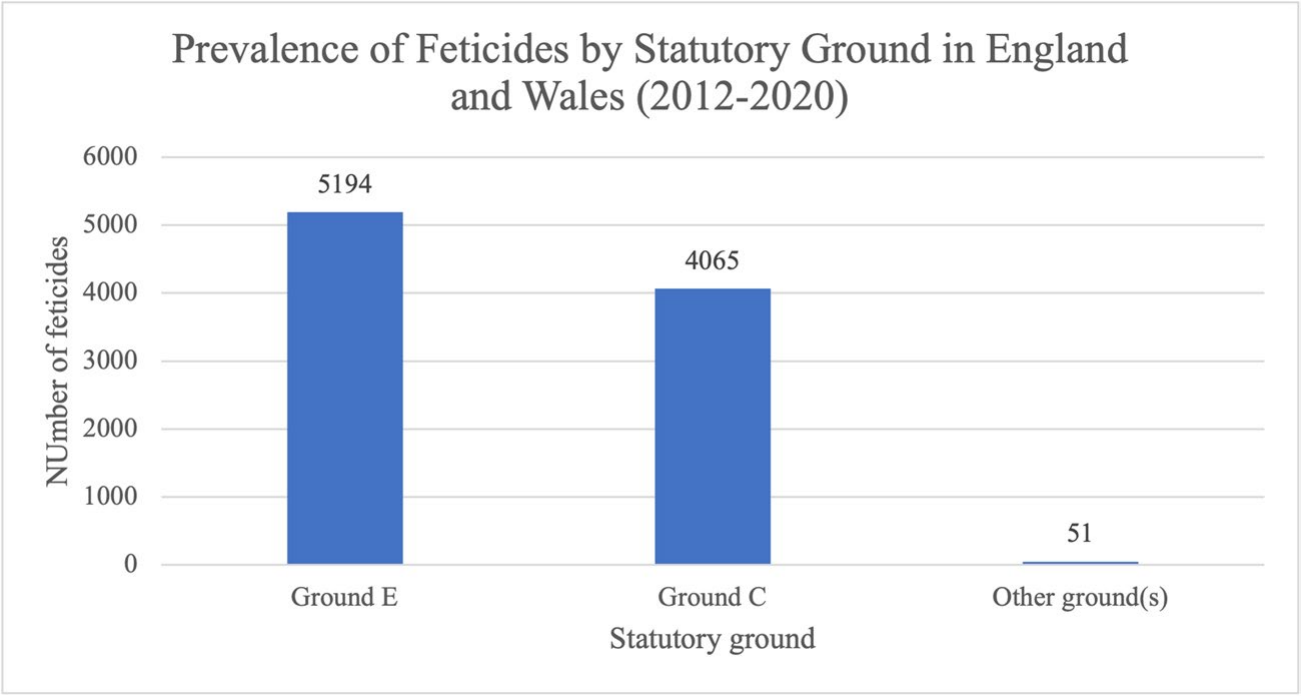
Prevalence of feticides by Statutory Grounds of the Abortion Act 1967 in residents and non-residents of England and Wales between 2012 and 2020. Data from the DHSC

Almost all abortions involving feticide in the study period were carried out under Ground C or E (Fig. 4); 5194 (55.8%) were carried out under Ground E, 4065 (43.6%) under Ground C and only 51 (0.6%) under other grounds, which could include Grounds A, B, D, F and G.

**Fig. 5:**
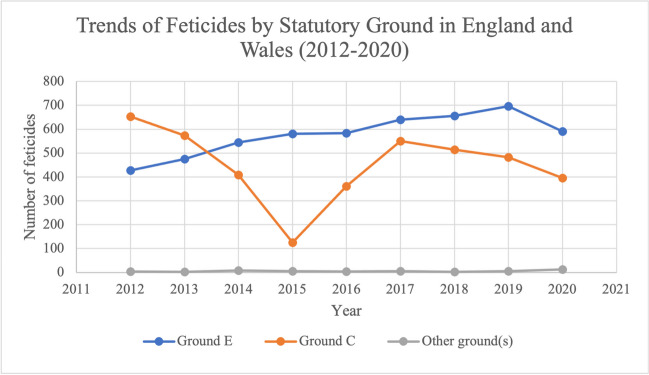
Trend over time of the number of feticides by Statutory Grounds of the Abortion Act 1967 in residents and non-residents of England and Wales between 2012 and 2020. Data from the DHSC

The number of feticides as part of terminations carried out under Ground E steadily increased from 2012 to 2019 by 269 (63.0%), from 427 cases in 2012 to 696 in 2019 (Fig. 5). In 2020, there were 591 feticides carried out under Ground E, a reduction of 15.1% from 2019. The changes in the number of feticides carried out under Ground C followed a similar trend as the overall number of feticides. Between 2012 and 2015, there was a steep decrease of 80.7%, from a peak of 653 cases in 2012 to a minimum of 126 in 2015. Between 2015 and 2017, there was an increase in feticides performed under Ground C. This steadily decreased again between 2017 and 2020, with 296 cases in 2020. Feticides carried out as part of terminations under other grounds (Grounds A, B, D, F and G) have remained consistently very low across the study period, ranging between 3 and 8 cases a year in 2012–2019 and then increasing slightly in 2020 to 13 feticides.

### Ground E Diagnosis

**Fig. 6:**
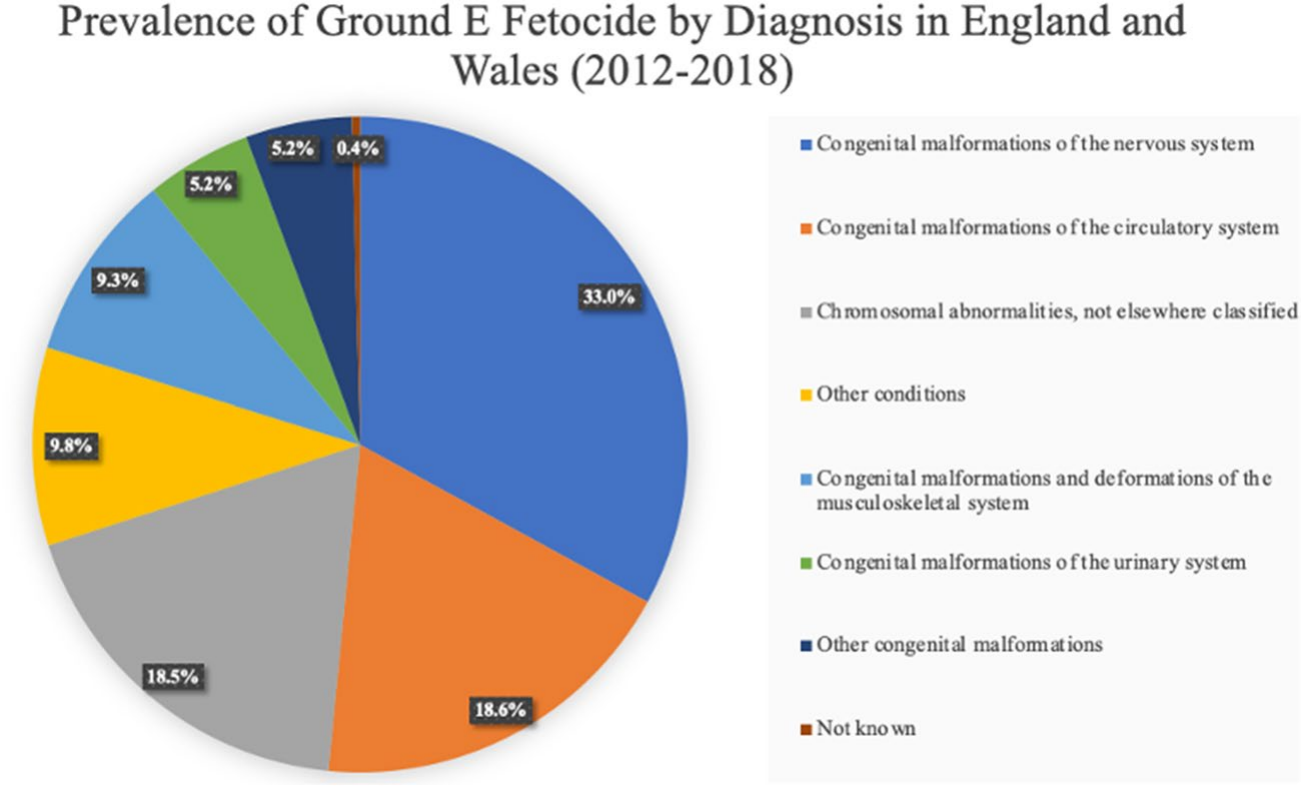
Prevalence of feticides under Ground E by primary diagnosis according to the systematic grouping of ICD-10 diagnostic codes in residents and non-residents of England and Wales between 2012 and 2018. Data from the DHSC

1288 cases (33.0%) of feticide performed under Ground E were due to a diagnosis of a congenital malformation of the nervous system, most frequently spina bifida. This was the most frequent systemic diagnosis, followed by congenital malformation of the circulatory system, which accounted for 726 feticides (18.6%) carried out under Ground E. This was closely followed by chromosomal abnormalities, which were the indication for 722 feticides (18.5%) performed under Ground E; 365 Ground E feticides (9.3%) were due to congenital malformations and deformations of the musculoskeletal system and 204 (5.2%) as a consequence of congenital malformations of the urinary system (Fig. 6).

Other congenital malformations were the reason for 205 (5.2%) feticides performed under Ground E. This includes diagnostic codes under the International Classification of Diseases (ICD-10) diagnostic category ‘other congenital malformations’ as well as cleft lip and cleft palate, congenital malformations of the respiratory system, other congenital malformations of the digestive system and congenital malformations of the eye, ear, face and neck; 134 of these cases were classified under ‘other congenital malformations’, and 25 or fewer cases were in each of the other four diagnostic categories; 381 (9.8%) Ground E feticides were due to the presence of other conditions which were not classified as congenital malformations or chromosomal abnormalities under ICD-10 criteria. These include a variety of conditions under ICD-10 diagnostic codes D10-D36, D37-D48, D55-59, E70-E90, G70-G73, O30-O48, P00-P04, P05-P08, P35-39, P50-P61, P80-P83, Z20-Z29 and Z80-Z99.

### Gestation

**Fig. 7:**
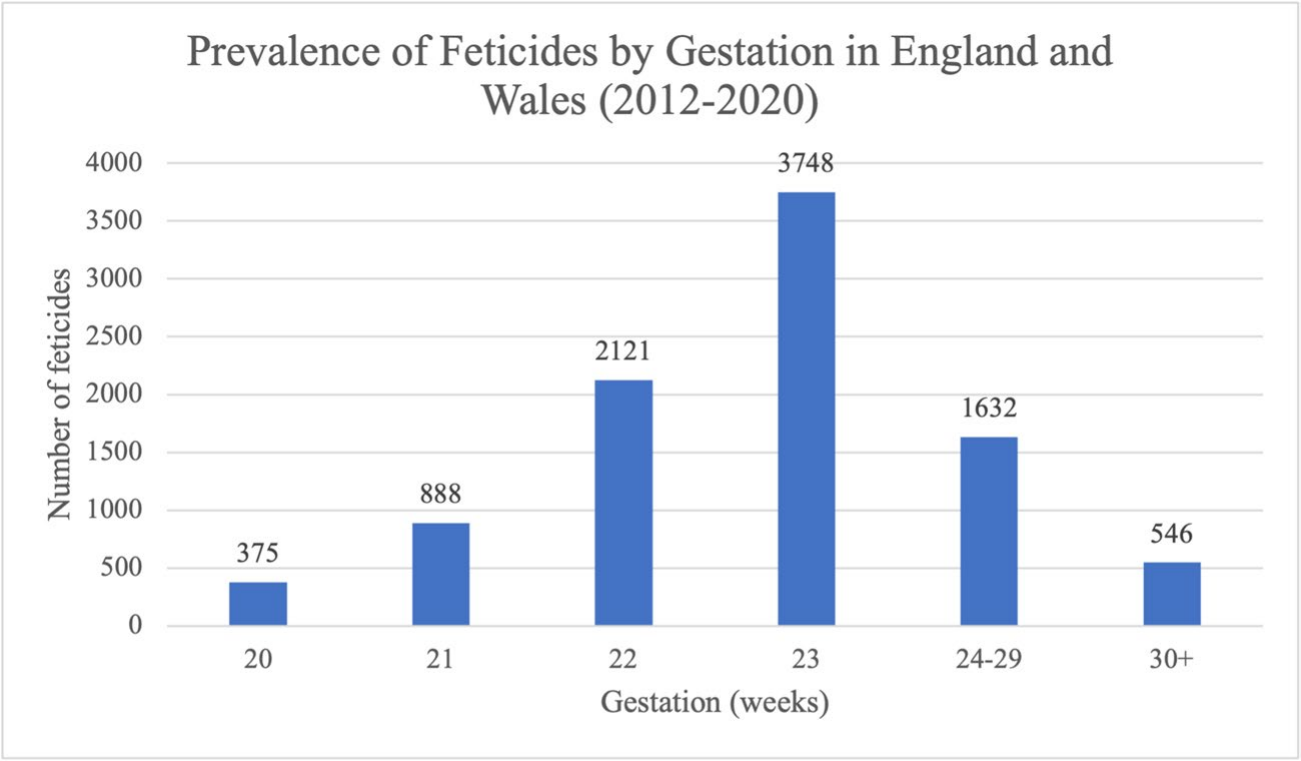
Prevalence of feticides by gestational age of the fetus in residents and non-residents of England and Wales between 2012 and 2020. Data from the DHSC

Feticide is recommended for terminations taking place after 21+6 weeks. The majority of feticide cases, 3748 (40.2%), took place at 23 weeks (Fig. 7). The subsequent most common gestation to perform feticide was 22 weeks, with 2121 feticides (22.8%) taking place at this gestational age. However, clinicians may also decide to use feticide earlier than the recommendation. This is reflected in the data as 1263 (13.5%) of feticides took place before 22 weeks, at 20 or 21 weeks. Termination is also legal after 24 weeks under Grounds A, B, E, F and G. During our study period, 1632 feticides (17.5%) took place between 24 and 29 weeks and 546 (5.9%) at 30 weeks or over.

### Provider

**Fig. 8:**
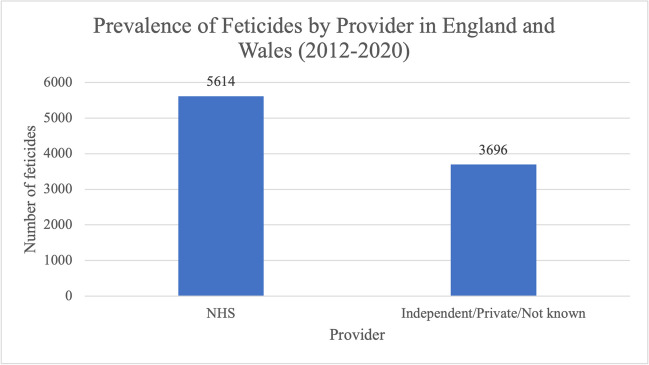
Prevalence of feticides by abortion provider in residents and non-residents of England and Wales between 2012 and 2020. Data from the DHSC

Abortions performed at 24 weeks gestation or over must be performed in an NHS hospital. Therefore, during the study period, more feticides took place in NHS hospitals than in independent clinics, private settings or those that were not known (Fig. 8); 5614 (60.3%) feticides took place in NHS hospitals; 3696 (39.7%) feticides were purchased privately and took place in independent clinics, or the provider was not known. Very few feticides took place in private hospitals or clinics. In almost all cases, private feticide at up to 24 weeks implies that they were purchased privately by the patient, who was most often a non-resident of England and Wales but took place in NHS hospitals or independent clinics. In less than 5 cases, the provider reported as unknown at the time the data was collated.

### Maternal Age

**Fig. 9:**
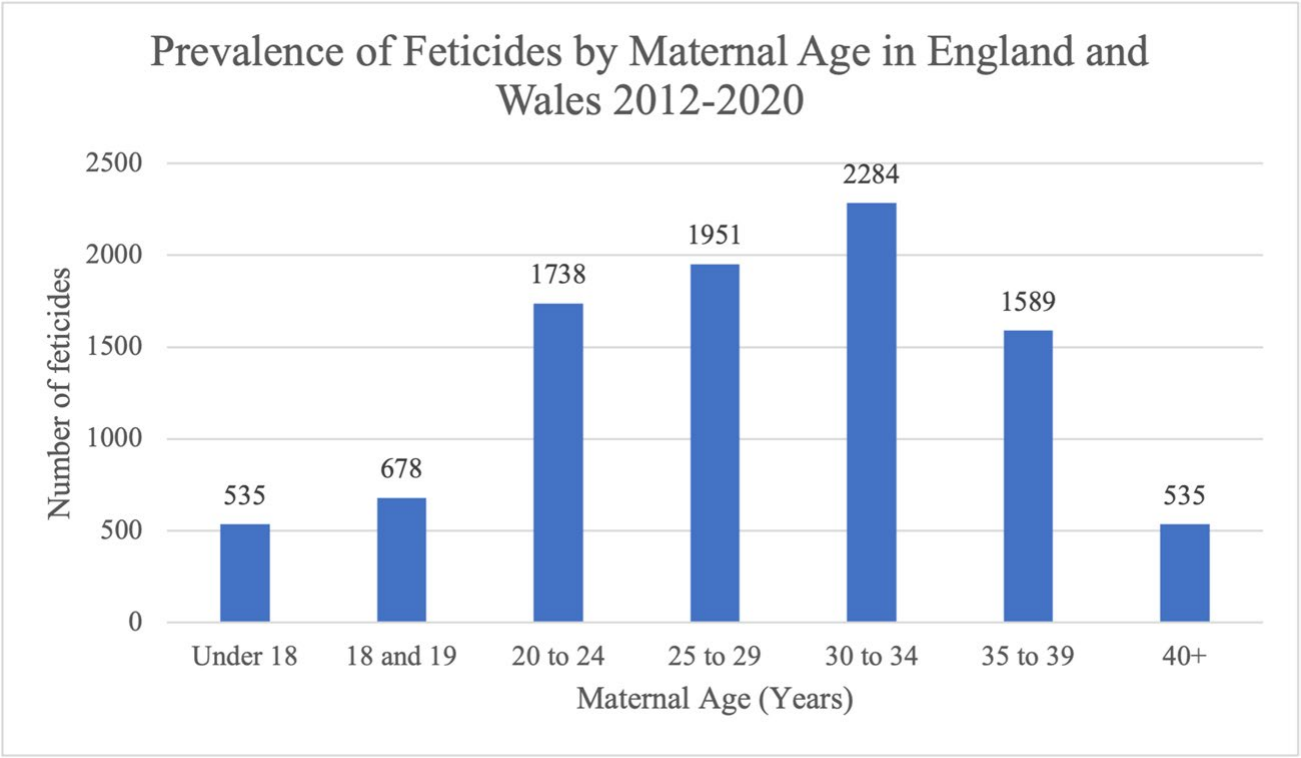
Prevalence of feticides by maternal age in residents and non-residents of England and Wales between 2012 and 2020. Data from the DHSC

Between 2012 and 2020, most feticides were carried out in women who were 30–34 years old; 3564 feticides (38.3%) occurred in this age group. A total of 1951 feticides (21.0%) were carried out in women aged 25–29 years. An additional 1738 (18.7%) in women aged 20–24 years and 1589 (17.1%) in women aged 35–39 years. Most, 81.2%, of feticides occurred in women aged 20–39 years, with 678 feticides (7.3%) being carried out for women aged 18 and 19 years. The number of feticides at the two extremes of the age groups, under 18 years and 40+ years, was the same, with 535 feticides (5.7%) occurring in each maternal age group (Fig. 9).

### Ethnicity

**Fig. 10:**
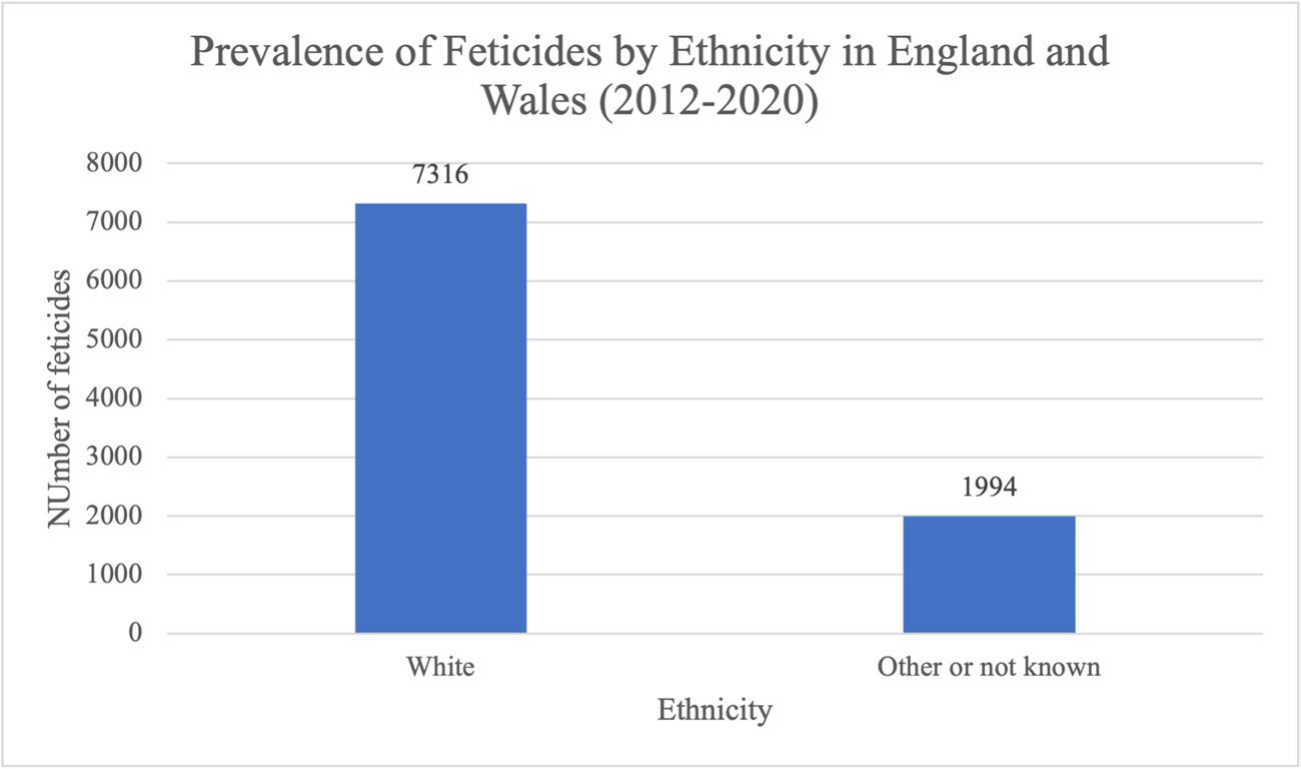
Prevalence of feticides by ethnicity in residents and non-residents of England and Wales between 2012 and 2020. Data from the DHSC

7316 (78.6%) feticides took place in women of white ethnicity (Fig. 10). The remaining ethnic backgrounds were not divided further in the data extraction; 1994 feticides (21.4%) took place in women whose ethnicity was classified as other or whose ethnicity was not known, in some cases because it was not stated.

### Obstetric History

**Fig. 11:**
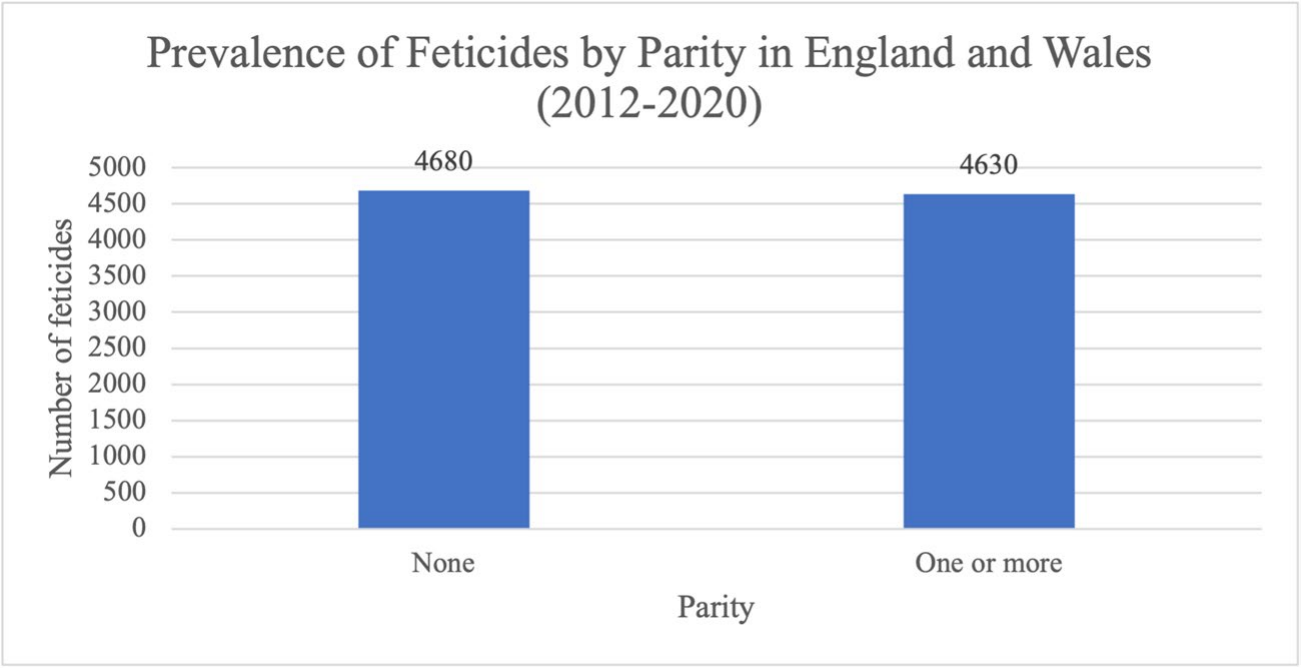
Prevalence of feticides by parity (number of previous liveborn or stillborn) in residents and non-residents of England and Wales between 2012 and 2020. Data from the DHSC

The number of feticides for the period 2012–2020 performed on women who had not previously given birth to a live born or stillborn (4680 (50.3%)) was almost the same as the number of women who had (4630 (49.7%)) (Fig. 11).

**Fig. 12:**
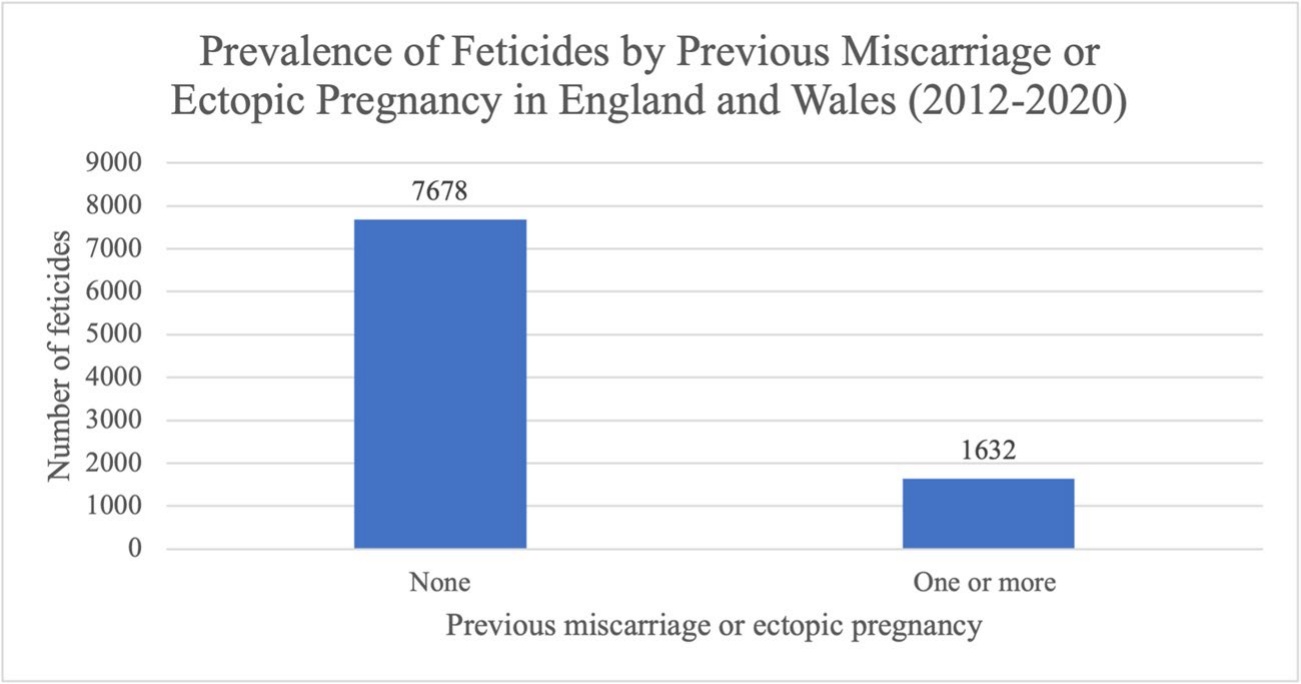
Prevalence of feticides by number of previous miscarriages or ectopic pregnancies in residents and non-residents of England and Wales between 2012 and 2020. Data from the DHSC

7678 women (82.5%) who underwent feticide did not have a previous miscarriage and/or ectopic pregnancy, compared to 1632 women (17.5%) who had experienced a miscarriage and/or ectopic pregnancy (Fig. 12).

**Fig. 13:**
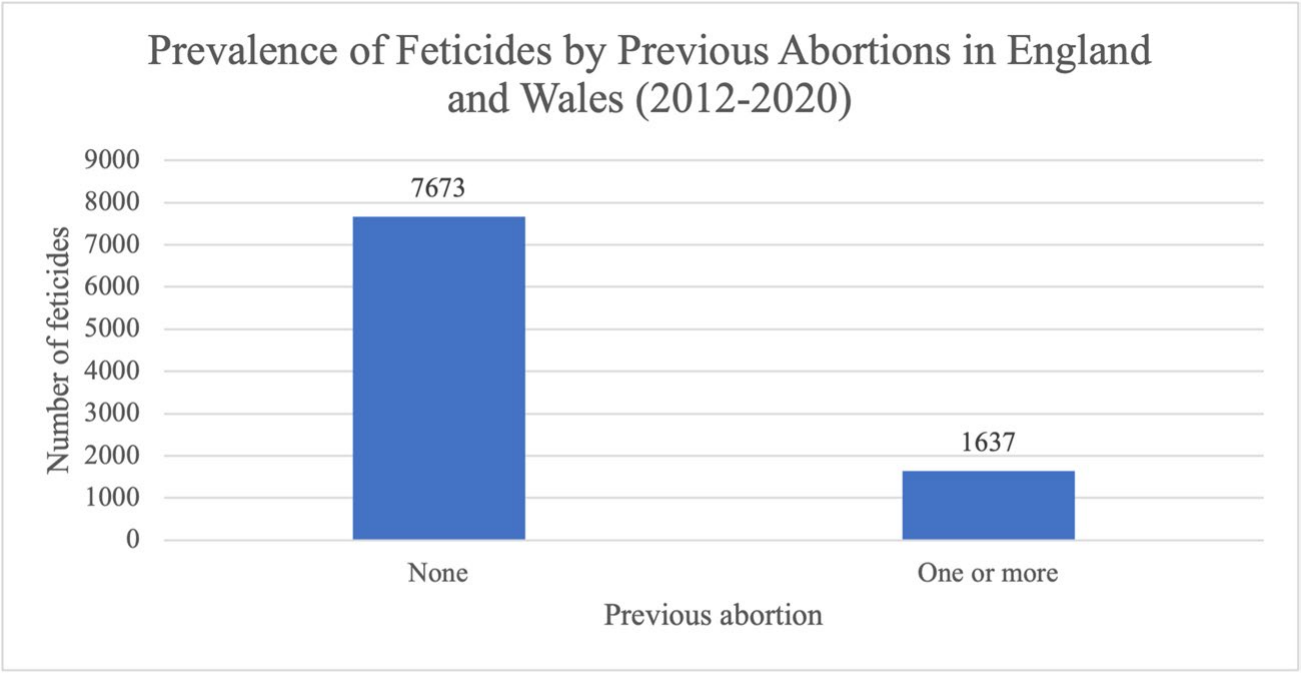
Prevalence of feticides by the number of previous abortions in residents and non-residents of England and Wales between 2012 and 2020. Data from the DHSC

For previous abortions, the results were very similar to previous ectopic pregnancies and/or miscarriages (Fig. 13); 7673 feticides (82.4%) took place in women without a previous abortion. The remaining 1637 feticides (17.6%) were in women who had previously terminated a pregnancy.

## Discussion

The study’s analysis of DHSC data over a 9-year period showed a fluctuating pattern of feticide, with a significant decrease in 2020 possibly tied to the COVID-19 pandemic, a finding consistent with other reports on reduced healthcare services during this period. An overall increase from 2012 to 2019 indicates a tendency for more terminations later in pregnancy. This aligns with a broader trend in the literature reflecting shifts in gestational timing of terminations, including findings from an Austrian hospital demonstrating increased late TOPs preceded by feticide [7].

The methods employed for feticide varied widely, with intracardiac KCl being the most prevalent. Other methods, such as lidocaine and digoxin, were also recorded. It was noted that certain alternative techniques were employed, even though evidence supporting their efficacy is scant [8–10].

Lidocaine was the second most common named method used for feticide. Research into the efficacy of lidocaine as a method of feticide has found a less than 100% success rate with intracardiac or umbilical lidocaine [8–10]. However, potential safety benefits of umbilical lidocaine over umbilical KCl have been suggested [10]. This may partially explain the decision to use lidocaine over KCl in these cases, despite the current guidelines in favour of intracardiac KCl.

Interestingly, cordotomy, intraamniotic KCl/urea, umbilical KCl/urea, extra amniotic KCl/urea and laser are currently being used, although rarely. This is despite very little evidence for their use in feticide and the availability of other options with a much greater evidence base. However, as with lidocaine, it is warned that it does not consistently induce fetal asystole [3]. This finding has been replicated in studies, primarily with regard to intraamniotic digoxin [11–13]. A potential explanation for why digoxin is still chosen in some cases over intracardiac KCl is that intraamniotic digoxin requires less technical skill than intracardiac KCl [14].

In terms of termination following feticide, more were medical than surgical. The distribution between Ground C and Ground E statutory grounds showed differences that were also reflected in gestational ages. This observation may be related to recent trends in prenatal screening, prenatal diagnosis technology and detection of congenital abnormalities over time as a result of an improvement in ultrasound screening skills [7]. The continual improvement in the prenatal diagnostic ability of congenital malformations is likely a contributing factor to the increase in feticides performed under Ground E.

A detailed analysis of Ground E diagnosis showed differences in primary indications, with congenital malformations more frequently reported for feticides, and chromosomal abnormalities for abortions. This distinction aligns with current screening practices and the timing of diagnoses.

The majority of feticides were carried out at 23 weeks, a finding that resonates with RCOG guidelines [3]. However, some were performed earlier, reflecting variations in global guidelines like those from the WHO and Austria. For example, the WHO recommends inducing fetal demise before abortion after 20 weeks gestation [15], and in Austria, feticide is used in TOPs taking place even earlier than 20 weeks [7]. Although studies have shown that most TOPs occur within 1–2 weeks of diagnosis [19], some of these later terminations may be due to delays in the time between diagnosis and termination. However, this is unlikely to explain such a high proportion of post-24-week terminations. A more likely reason is that some conditions are only detected when an ultrasound is performed after 24 weeks, for example, in the context of slow fetal growth, polyhydramnios or premature contractions [19].

Most feticides occurred in NHS hospitals, reflecting the specialist skills needed and independent or private clinics are registered to perform abortions for only up to 24 weeks [16]. The age distribution was notable for the predominance of women aged 30–34 years. It is well established that the risk of chromosomal abnormalities increases with maternal age. However, there is limited and mixed evidence on an association of increased maternal age with non-chromosomal anomalies, which were the primary diagnosis in over 70% of feticides in this study. It has been suggested instead that the risk of non-chromosomal malformations decreases with increasing maternal age. One study found that women aged 34 years or less had an increased risk of a major fetal anomaly compared with those aged 35 and over [17]. This, as well as the conception rate being by far the highest in women aged 25–34 in recent years [18], may explain the highest prevalence of feticide in this age group.

No significant differences in obstetric history were observed, and ethnicity appeared to be proportionate to overall abortion rates.

## Limitations and Future Research

The study’s retrospective nature and limitations with data coding and collection restricted the analysis of certain variables. Prospective studies and more detailed data collection could address these limitations.

Additionally, this study was limited in its analysis of the methods of feticide. Not all methods are coded separately, and thus, many fall under the category of Other, which was the second largest category of methods of feticide in this study. When HSA4 forms are submitted electronically by clinicians or data is uploaded by the DHSC from paper forms, not all methods of feticide have their own category. For example, in the years of this study, there was no separate option for digoxin, so if this was mentioned on a form, it would be coded as Other. However, the DHSC intends to improve this in future years to increase the detail of data on feticide. For example, in 2021, digoxin was added as its own category.

Unfortunately, due to the nature of the forms, there were no data collected by the DHSC on maternal analgesia, fetal anaesthesia, religion, maternal BMI, success rate or safety issues such as side effects. These can affect the safety and success rate of the procedure. Adequate maternal analgesia ensures the woman is comfortable and relaxed, which may aid in the precise feticide. This can enhance the success rate by ensuring the procedure is carried out meticulously. Fetal anaesthesia is often used to prevent potential fetal pain during the procedure. The proper administration may allow for a smoother process and possibly increase the success rate of the feticide.

Religious beliefs can affect the willingness of the patient to follow medical advice or cooperate fully during the procedure, impacting the success rate indirectly.

Religious objections might lead to delays or refusal of the procedure, resulting in increased health risks if the termination is medically necessary.

High or low maternal BMI might lead to technical difficulties in performing the feticide, such as visualising the fetus properly, potentially reducing the success rate.

A high BMI might be associated with other underlying health conditions like hypertension or diabetes, which can pose additional risks during the procedure. It might also affect the pharmacokinetics of drugs used, leading to complications. Conversely, low BMI might also indicate underlying nutritional deficiencies, which could lead to an increased risk of complications during or after the procedure.

It is worth mentioning that these are general considerations, and individual cases can vary widely. The involvement of skilled healthcare professionals who take all these factors into account and tailor the procedure to each patient’s specific needs and conditions is essential to optimise both the safety and success rate of feticide before the termination of pregnancy.

The presence of this additional data may have helped us to greater understand the reasons behind using the different methods of feticide. For example, methods used less frequently may have been associated with more side effects or safety issues and lower success rates.

As there are limitations with retrospective studies, a prospective study looking at feticide in England and Wales could provide additional insight. Despite clear recommendations from the RCOG on intracardiac KCl being the most reliable feticide method, only two-thirds of feticides in this study were performed using intracardiac KCl. Further qualitative research into clinicians’ experience performing feticide, preferred methods, and why could provide valuable insight. This could also provide a further understanding of how the procedure is carried out to support both patients and clinicians involved in feticide. Furthermore, as this is a national study, it would be interesting for similar research to be done in other countries using national data and comparing how feticide is performed globally. This can provide further awareness of how feticide is carried out in different cultures, with different attitudes and laws towards abortion, specifically at later gestations. This would allow a more profound understanding of the best practice of feticide and enable services to provide the highest quality recommendations and care for patients undergoing this emotionally complex clinical procedure.

## Conclusions

The study’s findings reveal significant insights into the trends and patterns of feticide in England and Wales, contributing to a broader understanding of this emotionally complex and clinically essential procedure. Comparative analysis with other research papers was limited by the specific data available, but the findings align with known trends and practices in related areas of reproductive health. Future research that builds upon these insights and incorporates international comparisons could further enrich our understanding and lead to improved patient care.

## Data Availability

Data is subject to third-party restrictions. The data supporting this study’s findings are available from The Department of Health and Social Care. Conditions apply to the availability of these data, which were used under license for this study. Data are available from the authors with the permission of The Department of Health and Social Care.
